# Asynchronous calibration of a CT scanner for bone mineral density estimation: sources of error and correction

**DOI:** 10.1093/jbmrpl/ziae096

**Published:** 2024-07-23

**Authors:** Alice Dudle, Michael Ith, Rainer Egli, Johannes Heverhagen, Yvan Gugler, Christina Wapp, Daniela A Frauchiger, Kurt Lippuner, Christian Jackowski, Philippe Zysset

**Affiliations:** ARTORG Center for Biomedical Engineering Research, University of Bern, sitem-insel, Freiburgstrasse 3, Bern 3010, Switzerland; Department of Diagnostic, Interventional and Pediatric Radiology, Inselspital, University of Bern, Freiburgstrasse 10, Bern 3010, Switzerland; Department of Diagnostic, Interventional and Pediatric Radiology, Inselspital, University of Bern, Freiburgstrasse 10, Bern 3010, Switzerland; Department of Diagnostic, Interventional and Pediatric Radiology, Inselspital, University of Bern, Freiburgstrasse 10, Bern 3010, Switzerland; ARTORG Center for Biomedical Engineering Research, University of Bern, sitem-insel, Freiburgstrasse 3, Bern 3010, Switzerland; ARTORG Center for Biomedical Engineering Research, University of Bern, sitem-insel, Freiburgstrasse 3, Bern 3010, Switzerland; ARTORG Center for Biomedical Engineering Research, University of Bern, sitem-insel, Freiburgstrasse 3, Bern 3010, Switzerland; Department of Osteoporosis, Inselspital, Bern University Hospital, University of Bern, Freiburgstrsasse 4, Bern 3010, Switzerland; Department of Osteoporosis, Inselspital, Bern University Hospital, University of Bern, Freiburgstrsasse 4, Bern 3010, Switzerland; Institute of Forensic Medicine, University of Bern, Murtenstrasse 28, Bern 3008, Switzerland; ARTORG Center for Biomedical Engineering Research, University of Bern, sitem-insel, Freiburgstrasse 3, Bern 3010, Switzerland

**Keywords:** quantitative CT, CT calibration, osteoporosis screening, BMD, bone imaging

## Abstract

The estimation of BMD with CT scans requires a calibration method, usually based on a phantom. In asynchronous calibration, the phantom is scanned separately from the patient. A standardized acquisition protocol must be used to avoid variations between patient and phantom. However, variations can still be induced, for example, by temporal fluctuations or patient characteristics. Based on the further use of 739 forensic and 111 clinical CT scans, this study uses the proximal femur BMD value (“total hip”) to assess asynchronous calibration accuracy, using in-scan calibration as ground truth. It identifies the parameters affecting the calibration accuracy and quantifies their impact. For time interval and table height, the impact was measured by calibrating the CT scan twice (once using the phantom scan with closest acquisition parameters and once using a phantom scan with standard values) and comparing the calibration accuracy. For other parameters such as body weight, the impact was measured by computing a linear regression between parameter values and calibration accuracy. Finally, this study proposes correction methods to reduce the effect of these parameters and improve the calibration accuracy. The BMD error of the asynchronous calibration, using the phantom scan with the closest acquisition parameters, was −1.2 ± 1.7% for the forensic and − 1.6 ± 3.5% for the clinical dataset. Among the parameters studied, time interval and body weight were identified as the main sources of error for asynchronous calibration, followed by table height and reconstruction kernel. Based on these results, a correction method was proposed to improve the calibration accuracy.

## Introduction

Quantitative analyses of BMDs with CT rely on a calibration method to convert CT numbers into BMD. Indeed, CT numbers are given in Hounsfield units (HUs), which, especially for dense bone tissue, vary for different scanners, scanning protocols, or patient sizes, for instance due to beam hardening.^1^ Correcting these variations is particularly important for estimating bone strength with finite element analysis since BMD differences can substantially impact strength values.^2-4^ The calibration procedure is based on reference materials with known density, such as a calibration phantom or, in the case of phantomless calibration, tissues present in the image.^1^^,^^5-8^ However, a typical limitation of phantomless methods is the low density of reference materials, for example, air, adipose tissue, aortic blood, muscle, which could lead to errors when extrapolated to cortical densities. To our knowledge, only one study so far has included cortical bone as reference material.^5^ On the other hand, calibration phantoms can include a broader range of density values. Although the phantom can be scanned with the patient (synchronous or in-scan calibration), this is rarely the case in clinical practice. Alternatively, the calibration relies on separate phantom scans (asynchronous calibration), whose scanning protocol should be as close as possible to the patient’s scan since parameters such as voltage considerably impact HU results.^9^ Yet, even with a standardized acquisition protocol, HU values may differ between the phantom and patient scans due to temporal variations in the X-ray source, manually set parameters (eg table height), or patient size.^10^ If these variations are not corrected in the asynchronous calibration procedure, they result in BMD errors, which can impact the diagnosis of bone diseases such as osteoporosis. The magnitude of such errors varies substantially, as described by the following studies.

For instance, the temporal stability of 7 scanners was analyzed by Engelke et al.^11^ They observed major fluctuations in 2 scanners after 1 yr, resulting in a BMD error increased by up to 8 times. Furthermore, the effect of resolution changes was evaluated by Troy et al., Eggermont et al., and Free et al., with differing results.^4^^,^^12^^,^^13^ Although the first 2 studies found that increasing the voxel size resulted in a decreased BMD (by 3%–4% and 1%–2% respectively), the third found no influence on BMD values.

Similarly, the impact of the scanner’s table height was studied by several authors, without showing any clear trend. According to Szczykutowicz et al., lower table height could result in increased or decreased HU values and noise by up to 72% and 130% respectively, depending on the body region.^14^ Toth et al. obtained an increase in noise by up to 30% for low positions.^15^ Brunnquell et al. showed that applying a calibration curve determined at the isocenter to a patient scanned 5 cm above or below resulted in a 5%–20% BMD error.^10^ The choice of the reconstruction kernel also impacts HU values, as shown by Michalski et al. and Giambini et al. Compared to a bone reconstruction kernel, a soft tissue image showed BMD values lower by 6.7% on average in the former study,^2^ and lower BMD (by 20%–25%) in the cortical but substantially higher BMD (by 2 to 57 times) in the trabecular bone in the latter.^16^ Finally, the effect of body size on measured bone densities was analyzed in several studies by adding layers of material around a phantom. L. Yu et al. obtained HU values reduced by 19%–38% for larger sizes,^17^ E. Yu et al. observed changes between –3.4% and +6.4% but no linear trend,^18^ and Bonaretti et al. reported scanner-dependent changes ranging from –9% to +5%.^19^

Considering the diverse and sometimes contradictory findings of these studies, we aimed to verify and extend these results. For this purpose, we used a forensic dataset that stands out by its large size (817 full-body scans), long collection duration (over 6 yr), and the inclusion of a calibration phantom with a broad density range (0–800 mg HA/cm^3^). To ensure the clinical relevance of our results, we combined this large dataset with a smaller clinical multi-scanner dataset.

We compared BMD values in the proximal femur (“total hip”) between asynchronous and in-scan calibration. We then computed the influence of various parameters on the BMD difference, such as body characteristics (weight, geometry), scanning parameters (voxel size, table height, reconstruction kernel), and time interval. Finally, we proposed a correction method for asynchronous calibration.

## Materials and methods

### Dataset

**Figure 1:**
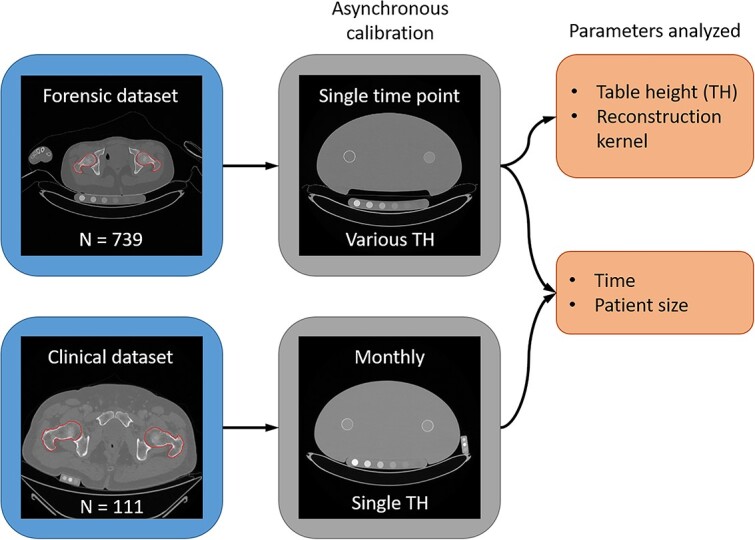
Overview of the datasets, calibration scans, and parameters analyzed.

The first dataset (called “forensic”) consists of 817 postmortem whole-body CT scans from the Institute of Forensic Medicine of the University of Bern, collected between 2015 and 2021 on a SOMATOM Definition AS 64 scanner (Siemens). The scans were performed as part of the autopsy procedure. Most scanning parameters were identical for all images, including the voltage (140 kV), the rotation time (0.5 s), the beam filter, and the automatic current modulation. Images were reconstructed iteratively with a slice thickness of 1 mm and an increment of 0.7 mm, using a bone (I70f\3) and a soft tissue (I31f\3) kernel. The CT scanner included a calibration phantom (QRM-BDC6 from QRM GmbH) integrated into the table pad, as illustrated in Figure 1 (upper left). The exclusion criteria for this study were metal implants or fractures in the hip region. The final set included 739 images.

Additional experiments were performed on the same scanner at the end of the collection period, with the calibration phantom in the scan table and a hip phantom (QRM HIP-QC120-Phantom from QRM GmbH) mimicking a patient (Figure 1, upper right). The phantoms were scanned at table heights ranging from 100 to 250 mm. Other scanning parameters were kept identical.

The clinical dataset consisted of 120 CT scans collected between 2021 and 2023 on 3 scanners at the Inselspital (Bern University Hospital). All participants provided written informed consent. Scans were performed for various medical purposes unrelated to the study and included a calibration phantom (custom-made with inserts from QRM GmbH), as illustrated in Figure 1 (lower left). Scanning and reconstruction parameters varied for each acquisition and are summarized in Table S1 in the Appendix, which also contains the scanners’ model. The calibration setup illustrated in Figure 1 (lower right) was scanned monthly on all 3 scanners, using the most frequent acquisition protocols at different voltages, which covered most of the parameter range shown in Table S1. Nine patient scans were excluded because their voltage did not match any of the reference phantom scans. The final set included 111 images.

Both study populations are described in Table 1. Both studies were approved by the local ethics commission (ID numbers 2019-01237 and 2022-00577).

**Table 1:** Description of the forensic and clinical datasets: sex, age, weight, and mean BMD in the proximal femur of the included subjects.

	Sex		Age [yr]			Weight [kg]			BMD [mg/cm^3^]		
	W	M	Mean	Min	Max	Mean	Min	Max	Mean	Min	Max
**Forensic**	235	504	52.9	13	94	78	29	138	412.9	148.8	612.2
**Clinical**	86	25	72.7	65	89	81	49	148	287.0	165.2	429.7

### Calibration

The CT images were calibrated with a synchronous method using the in-scan phantom and with an asynchronous method using the phantoms scanned separately. The same phantom was used within each study for in-scan and asynchronous calibration: a phantom with 6 inserts containing hydroxyapatite (HA) concentrations ranging from 0 to 800 mg/cm^3^ for the forensic dataset and a phantom with 3 inserts ranging from 300 to 800 mg HA/cm^3^ for the clinical dataset. Inserts were located with a circle detection algorithm based on the Hough transform.^20^ The detected radii were reduced by 20% to avoid partial volume effects before averaging the HU values for each insert. A linear regression was computed between the average HU values and the true HA densities and used to convert the original CT values into BMD.

In the forensic dataset, asynchronous phantom measurements were only available for one time point, but at different table heights. For the asynchronous calibration, scans were calibrated twice, with different reference phantom scans. The first asynchronous calibration used the same reference scan for all cases, with a table height in the middle of the possible range (160 mm). The second asynchronous calibration adapted the reference scan according to the actual table height based on a personalized interpolated calibration curve. The interpolation procedure with a piecewise linear curve is described in Appendix 4.

In the clinical dataset, monthly asynchronous phantom measurements were available for each scanner and for up to 12 common acquisition protocols. The reference scan for the asynchronous calibration was chosen with the following criteria, in that order: (1) same scanner, (2) most similar protocol (in particular, same voltage), and (3) closest time point. To quantify the effect of the time interval, a second asynchronous calibration was computed using the same reference phantom scan for all cases (last available time point) instead of the phantom scan closest in time. The image processing was implemented in Python 3.6 and based on functions from the SimpleITK (version 2.0) and OpenCV (version 4.5) libraries.

### Segmentation

The calibration comparison focused on BMD values in the proximal femur, therefore a segmentation mask had to be defined for this region. To obtain more accurate results in the acetabulum region, both pelvis and proximal femur were segmented. Each region was manually segmented in 18 images, which were then used to train a neural network based on the nnU-Net^21^ method. The resulting segmentation mask contained the proximal region of both femurs and was used to obtain the average BMD value in that region, illustrated in Figure 1 (left) and in Figures S1 and S2.

### Statistical analysis

#### Asynchronous calibration accuracy

BMD values obtained with in-scan and asynchronous calibration were compared by computing a linear regression. Using the in-scan calibration as ground truth, the BMD error of the asynchronous calibration was computed as follows:


$$ d=\frac{\left( BM{D}_{asyn}- BM{D}_{inscan}\right)}{BM{D}_{inscan}} $$

#### Table height and time interval

Table height is defined as the distance between the scanner’s isocenter and the top of the scanning table and is positive below the isocenter. The effect of table height on the calibration accuracy was assessed in the forensic dataset by comparing the BMD error of the 2 asynchronous methods described above, with fixed and personalized table height. Similarly, the effect of the time interval on the calibration accuracy was measured on the clinical dataset by comparing the BMD error obtained for an asynchronous calibration with a reference scan at a fixed time point and with the closest available time point. For both parameters, the BMD difference was tested for significance with Student’s *t*-test for paired samples.

#### Patient and acquisition parameters

For the remaining parameters, the correlation with the BMD error was computed and tested for significance with Student’s *t*-test. The parameters of interest were first identified in the forensic dataset, based on its large size and more constant scanning parameters. The parameters tested included body weight, BMI, body volume, polar moment of area, and voxel size. Body volume (average per slice) and polar moment in the hip region were obtained by segmenting the body surface with Otsu’s method.^22^ The obtained relationships were then verified in the clinical dataset.

#### Reconstruction kernel

In the forensic dataset, the above analysis was performed for the bone kernel image, generally preferred for BMD analysis. Indeed, sharper edges facilitate the detection of bone surfaces and reduce partial volume effects. However, soft tissue reconstruction is sometimes also used, especially in opportunistic analyses. Therefore, BMD values obtained with the bone and soft tissue kernels were compared as a last step. The soft kernel images were calibrated asynchronously, using the same method as for bone kernel images. The BMD difference between both reconstructions was recorded and tested for significance with Student’s *t*-test for paired samples.

## Results

### Asynchronous calibration accuracy

**Figure 2:**
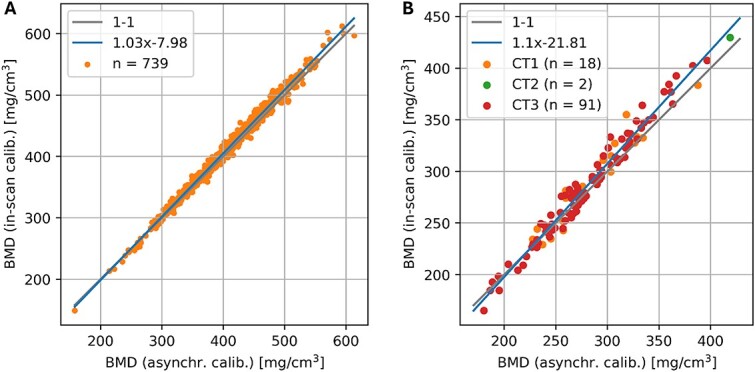
Relationship between proximal femur BMD values computed with an in-scan and asynchronous calibration method in the forensic (A) and clinical (B) dataset.

In the forensic and clinical datasets, the asynchronous and in-scan calibrations showed a very good correspondence, with *R*^2^ = 0.99 and 0.97, respectively, as illustrated in Figure 2. In the clinical dataset, BMD errors observed in the 3 CT scanners were not significantly different from each other, as shown in Table S2.

### Table height

The forensic dataset was calibrated asynchronously with calibration curves corresponding to a fixed and a personalized table height, respectively. According to a paired *t*-test, BMD values were not significantly different between both methods. Nonetheless, the SD and maximal absolute error are slightly reduced for the personalized table height, as shown in Table 2.

**Table 2:** BMD errors obtained with a fixed and personalized table height (TH) for the asynchronous calibration of the forensic dataset.

	Mean [%]	SD [%]	Abs. Max. [%]	*R* ^2^
**Fixed TH**	−1.1	2.0	8.7	0.99
**Personalized TH**	−1.2	1.7	6.1	0.99

The coefficient *R*^2^ corresponds to the correlation between asynchronous and in-scan calibration.

### Time interval

**Figure 3:**
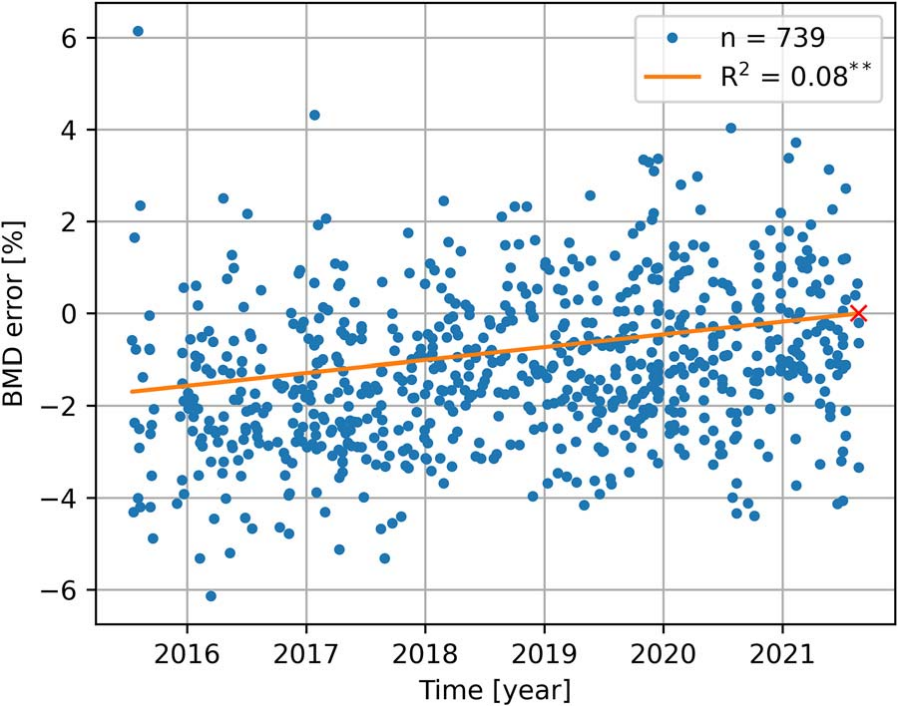
Temporal evolution of the BMD error in the forensic dataset, with linear regression (^*^^*^p<.01). The time point of the phantom scan is illustrated by the cross.

For the forensic dataset, the asynchronous phantom measurements were only performed at one time point, at the end of the collection period. To quantify temporal variations, we computed a linear regression between the BMD error and the time interval between phantom and donor scans. As shown in Figure 3, the BMD error is minimal close to the phantom scan and increases by 0.28% per year.

The clinical dataset was calibrated asynchronously using either the last or the closest calibration measurements to evaluate variations with time. The same acquisition protocol was used for both phantom measurements. As shown in Table 3, using the closest time reduces the mean BMD error significantly but does not affect the SD.

**Table 3:** BMD errors obtained using a fixed or the closest time point for the asynchronous calibration.

	Time interval [d]		BMD error [%]			*R* ^2^
	Mean	Max.	Mean	SD	Abs. max.	
**Fixed time**	404	856	−3.5	3.4	11.4	0.97
**Closest time**	11	33	−1.6	3.5	10.3	0.97

The coefficient *R*^2^ corresponds to the correlation between asynchronous and in-scan calibration.

### Patient and acquisition parameters

Table 4 shows the correlation between the BMD error and various patient and scanning parameters in the forensic dataset. All correlations were significant, with the largest correlation coefficient for body weight. Since all other parameters correlated significantly with body weight, we decided to keep only that parameter in the following analyses.

**Table 4:** Correlation of individual parameters with the BMD error.

	Correlation coefficient (*R*^2^)
**Average slice volume**	0.52^a^
**Body weight**	0.57^a^
**BMI**	0.46^a^
**Polar moment**	0.01^a^
**Voxel size**	0.32^a^

a *p*<.01

**Figure 4:**
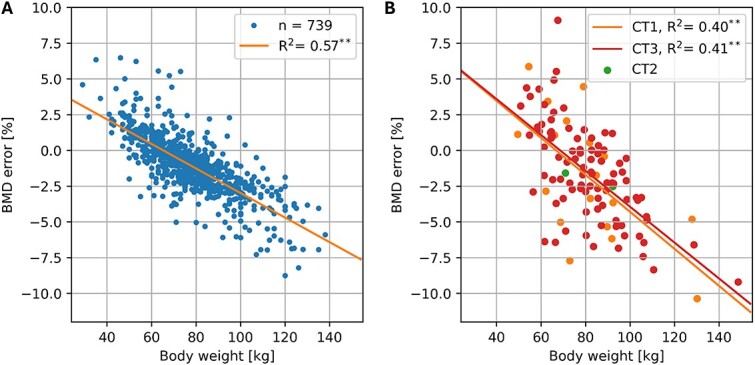
BMD error of the asynchronous calibration as a function of body weight for the forensic (A) and clinical (B) datasets (^*^^*^p<.01).

A linear regression between body weight and BMD error was fitted for each scanner independently, except for CT2, which had only 2 observations. Correlation coefficients and regression equations are recorded in Table 5 and illustrated in Figure 4.

**Table 5:** Parameters of the regression between body weight and BMD error, and BMD error after correction for the body weight, for the clinical and forensic (fixed table height) CT scanners.

	*R* ^2^	Regression equation	BMD error [%]		
			Mean	SD	Abs. max.
**CT1**	0.40^a^	8.66–0.13x	−0.11	2.89	7.0
**CT3**	0.41^a^	8.62–0.13x	−0.07	2.62	9.0
**Forensic CT**	0.57^a^	5.61–0.09x	0.02	1.34	6.3

a *p*<.01

The regression equation was significantly different between forensic and clinical scanners but not between clinical scanners CT1 and CT3. Interestingly, the root of all 3 equations lies between 62 and 67 kg, which is a realistic body weight.

### Reconstruction kernel

A significant BMD difference of 5 ± 1% was observed in the forensic dataset between soft and bone reconstruction kernels, as reported in Table 6. These results could not be verified on the clinical dataset, as each scan was reconstructed only once.

**Table 6:** Proximal femur BMD estimated from a bone (I70) and a soft tissue (I31) kernel reconstruction.

	BMD [mg/cm^3^]	
	Mean	SD
**Bone kernel**	408	65
**Soft tissue kernel**	386	63
**Difference**	21^a^	3

a *p*<.01

### Correction

Using in-scan calibration as ground truth, we propose a correction method for asynchronous calibration. The effect of the correction method on the BMD error is illustrated in Figure S4 in the Supplementary Materials.

First, we showed that reducing the time interval between the patient and reference phantom scan decreased the BMD error by more than 50%. Therefore, we suggest performing phantom measurements at regular intervals, for example, monthly, on each CT scanner of interest for common acquisition protocols. Indeed, other scanning parameters, such as voltage, impact the calibration accuracy^9^ and should be identical in phantom and patient scans.

Second, adapting the table height of the phantom scans to the patient value reduced the SD of the BMD difference by 15%, avoiding the largest differences. Therefore, we suggest repeating phantom measurements at several table heights and interpolating the results to obtain reference values for any table height. As this procedure might be considered too time-consuming for a monthly repetition, the table measurement could instead be performed once, and the resulting correction curve extrapolated to other time points. Since table height variations are likely to be scanner-dependent, measurements should be repeated on each scanner of interest.

Furthermore, body weight correlated significantly with BMD error. To quantify these variations, commercially available extension rings can be combined with the calibration phantom to simulate different patient sizes.^23^ However, if only one phantom size is available, the impact of body weight cannot be quantified with phantom measurements. Instead, we suggest computing a linear regression between body weight ($m$) and BMD error ($d$) for a set of in-scan calibrated patients, as done in this study: $d=a\cdot m+b$. This regression is probably scanner-dependent, as shown by the different regression curves. Using parameters $\left(a,b\right)$, a body weight correction can be integrated into the asynchronous calibration procedure:


$$ BM{D}_{asyn}^{corr}= BM{D}_{asyn}\left(1-a\cdot m-b\right) $$

Applying this correction to the BMD values from the forensic and clinical (CT1 and CT3) scanners reduced the mean BMD error by 93%–98% and the SD by 17%–33%.

Finally, the reconstruction kernel impacts the BMD outcome. Therefore, it is crucial to use the same kernel within a study or to choose a kernel of reference and compute a correction factor for the other kernel(s), for example, on a subset of patients reconstructed with different kernels. As the kernels vary between manufacturers and CT models, the correction factor should be computed for each pair of kernels.

## Discussion

This study aimed to identify parameters that are not standardized in the acquisition protocol but impact the accuracy of asynchronous calibration. Using forensic and clinical datasets, we found that time, table height, body weight, and reconstruction kernel were responsible for BMD errors.

The impact of the time interval on asynchronous calibration accuracy has been studied by Engelke et al., who found a stable BMD accuracy in 5 scanners, while 2 showed an increased BMD error (by up to 8 times) after 1 yr.^11^ In comparison, our clinical scanners showed intermediate stability with a BMD error increased by a factor of 2 when using a distant time point. These temporal variations could be due to the X-ray source evolution over time, or to hardware and software upgrades^11^ and underline the need for regular calibration measurements.

The forensic dataset showed remarkable temporal stability over 6 yr. Although a single phantom measurement was performed at the end of the collection period, the BMD error between in-scan and asynchronous calibration increased by only 0.28% per year. In comparison, the short-term precision found by Bligh et al. was 1.4%.^24^ The fixed acquisition protocol, for instance, the constant voltage and reconstruction parameters, is likely beneficial to the temporal stability and creates a homogeneous dataset for research.

Using a personalized table height in the asynchronous calibration allowed to reduce the SD of the BMD error by 15%. However, this effect could not yet be verified in clinical data. Interestingly, the phantom measurements showed a piecewise linear relationship between table height and HU values. This might partly be caused by the bowtie filter, designed to distribute the dose more homogeneously but with unexpected effects if the body is misplaced.^14^^,^^15^ For very low table heights, part of the phantom might also be out of the field of view, resulting in truncation artifacts. Most previous studies did not examine table heights lower than 100 mm and would thus be expected to report only the decreasing part of the curve. In the study by Szczykutowicz et al., this is the case for anterior regions in the body but not for posterior ones.^14^

The BMD error correlated significantly with body weight. Larger body weights resulted in lower BMD values, which is in line with L. Yu et al.^18^ This effect is probably due to beam hardening,^10^ which affects asynchronous more than in-scan calibration accuracy. Indeed, E. Yu et al.^17^ did not observe a linear trend between body size and BMD when using an in-scan phantom for the calibration. The relationship might also be scanner-dependent, as indicated by Bonaretti et al.^19^ and by the different regression equations obtained.

After correcting for body weight, mean BMD errors practically vanished. However, it is important to consider that using the regression method proposed here might also correct for other systematic errors between asynchronous and in-scan calibration unrelated to body weight. Therefore, the effect of body weight on BMD errors should not be overestimated based on the mean error reduction. Nonetheless, the SD of the error is also reduced by 17%–33%, which might be a more realistic estimate of the body weight impact.

The effect of other parameters related to body size, such as body volume, BMI, or polar moment of area, was less strong and became insignificant after correcting for body weight. This is not surprising, as each of these parameters was strongly correlated with body weight.

In addition, we observed a significant BMD difference (–5.3%) between soft and bone reconstruction kernels. This is close to the result observed by Michalski et al. (−6.7%)^2^ but substantially smaller than the values reported by Giambini et al., who obtained up to 25% BMD difference in the cortical bone.^16^ However, they reported results for cortical and trabecular bone separately, while our region of interest contains both.

Finally, the worst-case BMD error for the asynchronous calibration with fixed table height and time was −3.5 ± 3.4% in the clinical dataset. Although this might be negligible in many clinical applications, the error could increase exponentially in the context of finite element analysis, impacting the resulting bone strength.^2^^,^^4^ Nonetheless, it shows the feasibility of estimating BMD from routine clinical CT scans. In the future, such estimations could be applied to any routine CT scans for opportunistic osteoporosis screening to identify patients at increased fracture risk without additional radiation.

The phantom presence was not investigated in this study but might have an impact on the BMD values due to the additional beam hardening. This has been shown previously to have a small, significant, but clinically negligible effect on the BMD results.^25^

When correcting BMD errors is impossible due to missing data for the relevant scanner, phantomless calibration can be a promising alternative. Using neighboring tissues as reference calibration materials has the advantage of correcting variations directly. This method has been studied in several publications,^1^^,^^5-8^ but is not yet widely used. A typical limitation is the density of the reference materials, which is substantially lower than cortical bone.

This study focused on BMD values in the proximal femur. Although the impact of the time interval might be similar in all body regions, the relationships observed for other variables should be verified for bones and body regions with a different composition and geometry, such as the foot or the wrist.

## Conclusions

Among the parameters studied in this work, time interval, body weight, table height, and reconstruction kernel were identified as the main sources of error for asynchronous calibration. Therefore, it is crucial to correct the variations produced by these parameters during the calibration to obtain accurate BMD values.

## Supplementary Material

Supplementary_materials_clean_ziae096

## Data Availability

The data will be made available upon request.
