# Supplementary material for: Asynchronous calibration of a CT scanner for bone mineral density estimation: sources of error and correction
Source: JBMR Plus. 2024 Jul 23;8(9):ziae096. doi: 10.1093/jbmrpl/ziae096 (PMC11344033; doi:10.1093/jbmrpl/ziae096)
Supplement: Supplementary_materials_clean_ziae096 [file supplementary_materials_clean_ziae096.docx]

Supplementary materials

1. Region of interest


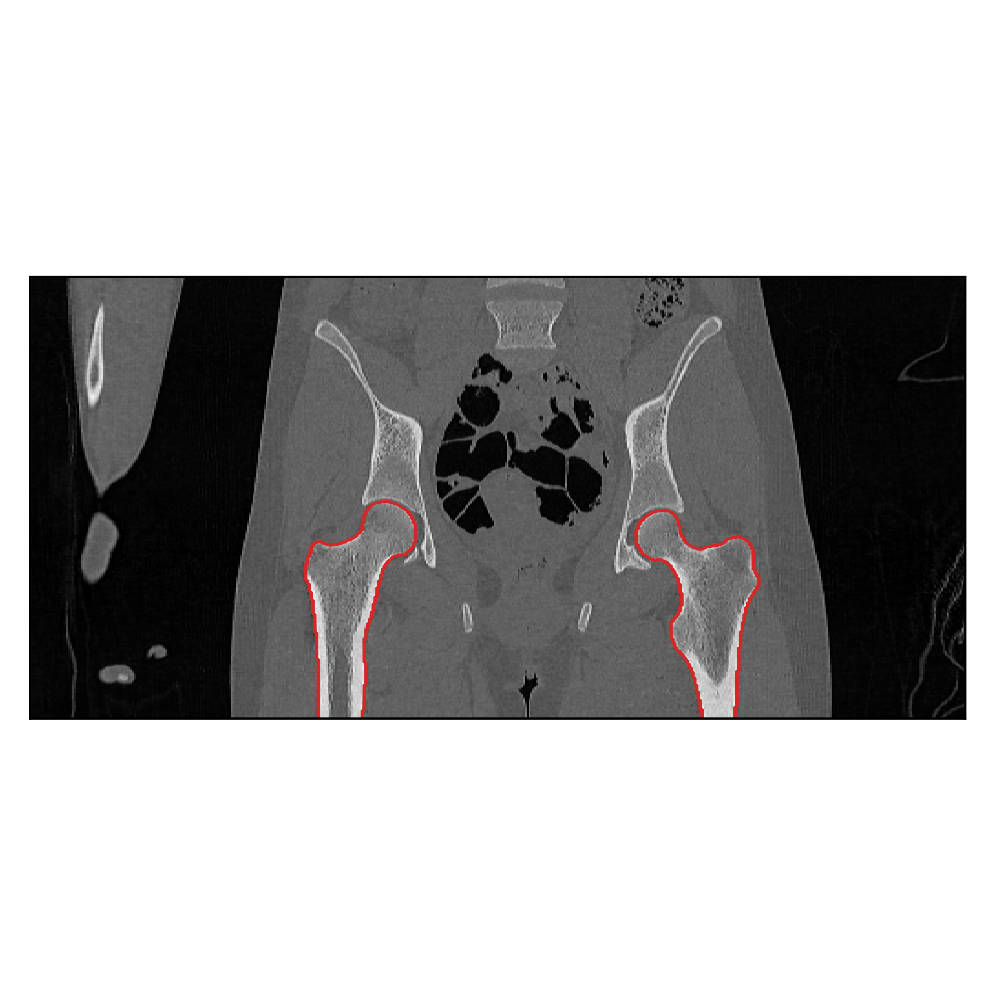


Supplementary Figure 1: Example of a CT image from the forensic dataset with the segmentation mask for the proximal femur region.


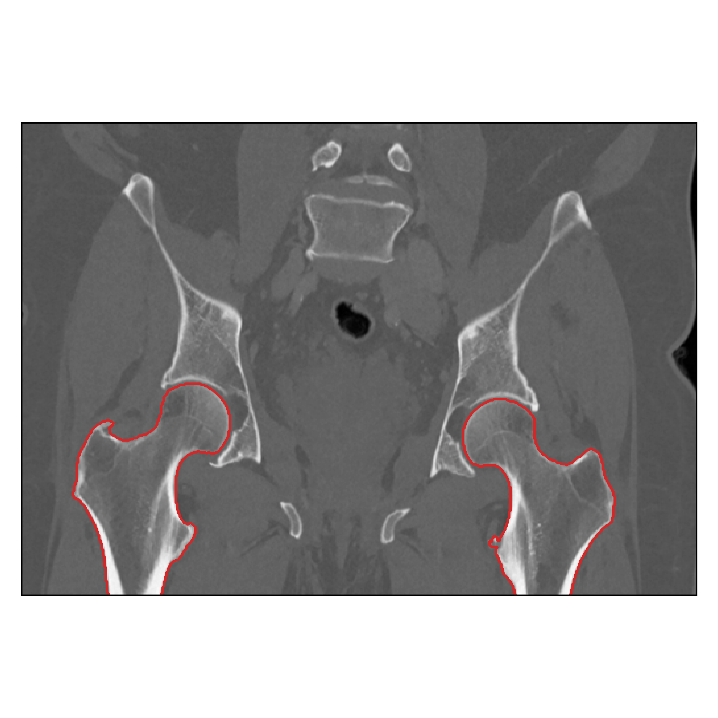


Supplementary Figure 2: Example of a CT image from the clinical dataset with the segmentation mask for the proximal femur region.

1. Scanning parameters
   1. Forensic dataset

The images were collected on a SOMATOM Definition AS 64 scanner (Siemens, Munich, Germany), with software version syngo CT 2012B. All scans were acquired with a voltage of 140 kV and a pitch factor of 0.5. The in-plane spacing ranged from 0.80 to 1.52 mm. Table height, positive when the table is below the isocenter, varied between 101 and 238 mm, with an average of 156 mm and a vast majority of scans (586) performed at 152.5 mm.

- 1. Clinical dataset

The scanning parameters of the clinical dataset are described in Supplementary Table 1.

Supplementary Table 1: Scanning parameters for the clinical dataset.

|  | | CT1 | CT2 | CT3 | All |
| --- | --- | --- | --- | --- | --- |
| Manufacturer | | Siemens | Siemens | Philips |  |
| Model | | Somatom Definition Flash | Somatom Force | Brillance 64 |  |
| Software | | syngo CT VA48A | syngo CT VB20A | Version 4.1 |  |
| Number | | 18 | 2 | 91 | 111 |
| Voltage | 100 kV | 16 | 1 | 0 | 17 |
|  | 120 kV | 2 | 1 | 91 | 94 |
| Kernel | Soft | 18 | 1 | 89 | 108 |
|  | Bone | 0 | 1 | 2 | 3 |
| Pitch | Min | 0.6 | 0.6 | 0.8 | 0.6 |
|  | Max | 0.6 | 1 | 1.42 | 1.42 |
| Spacing [mm] | Min | 0.74 | 0.74 | 0.68 | 0.68 |
|  | Max | 0.98 | 0.74 | 0.98 | 0.98 |
| Table height [mm] | Min | 130 | 167.5 | 65.3 | 65.3 |
|  | Max | 199 | 185.5 | 161.5 | 199 |

1. Comparison between scanners

The BMD error of the asynchronous calibration in the three clinical scanners is reported in Supplementary Table 2.

Supplementary Table 2: BMD error in the three clinical scanners.

|  | N | BMD error [%] | | |
| --- | --- | --- | --- | --- |
|  |  | Mean | Std. dev. | Abs. max. |
| CT1 | 18 | -1.8 | 4.4 | 10.3 |
| CT2 | 2 | -2.0 | - | 2.5 |
| CT3 | 91 | -1.6 | 3.4 | 9.2 |

1. Table height regression

The phantom measurements were repeated at various table heights and interpolated to obtain calibration curves at any table height. Visual inspection showed that a linear relationship was not appropriate for this variable. Instead, a piecewise linear regression with two segments was fitted for each insert, as illustrated in Supplementary Figure 4 for insert 1, and described in the following equation:

$$H^{i}\left( t \right)=\left\{ \begin{aligned} H_{b}^{i}+s_{1}^{i}\left( t-t_{b} \right) ; t\leq t_{b} \\ H_{b}^{i}+s_{2}^{i}\left( t-t_{b} \right) ;t>t_{b} \end{aligned} \right.$$

where $H^{i}\left( t \right)$ is the HU value for insert $i$ at table height $t$, $t_{b}$ and $H_{b}^{i}$ are the table height and HU value at the breakpoint between both segments, $s_{1}^{i}$ and $s_{2}^{i}$ are the slopes of each segment. For every breakpoint position $t_{b}$ in the table height range, $s_{1,}^{i}s_{2,}^{i}H_{b}^{i}$ were optimized simultaneously for each insert, while $t_{b}$ was fixed. The root mean square error (RMSE) between predicted and observed HU values was then computed and averaged between all inserts for each breakpoint position. One optimal breakpoint position $t_{b}$ was then chosen for all inserts based on the lowest mean RMSE.


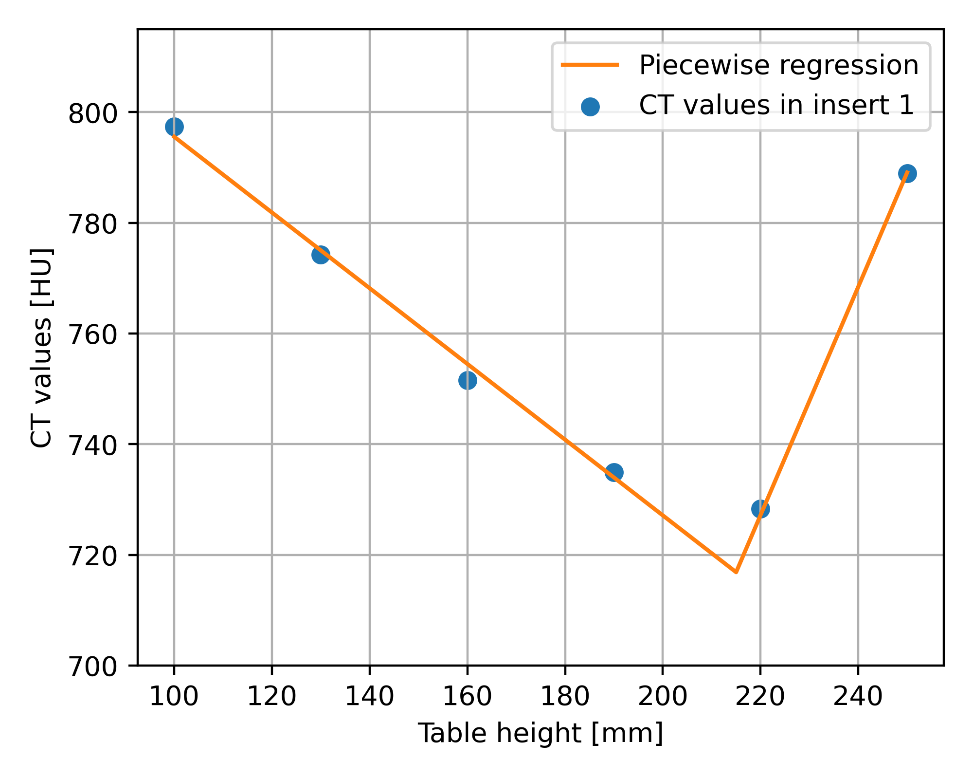


Supplementary Figure 3: HU variations in insert 1 (800 mg/cm^3^) as a function of the table height with a piecewise linear regression. HU values in other inserts showed similar behaviors.

1. Correction method


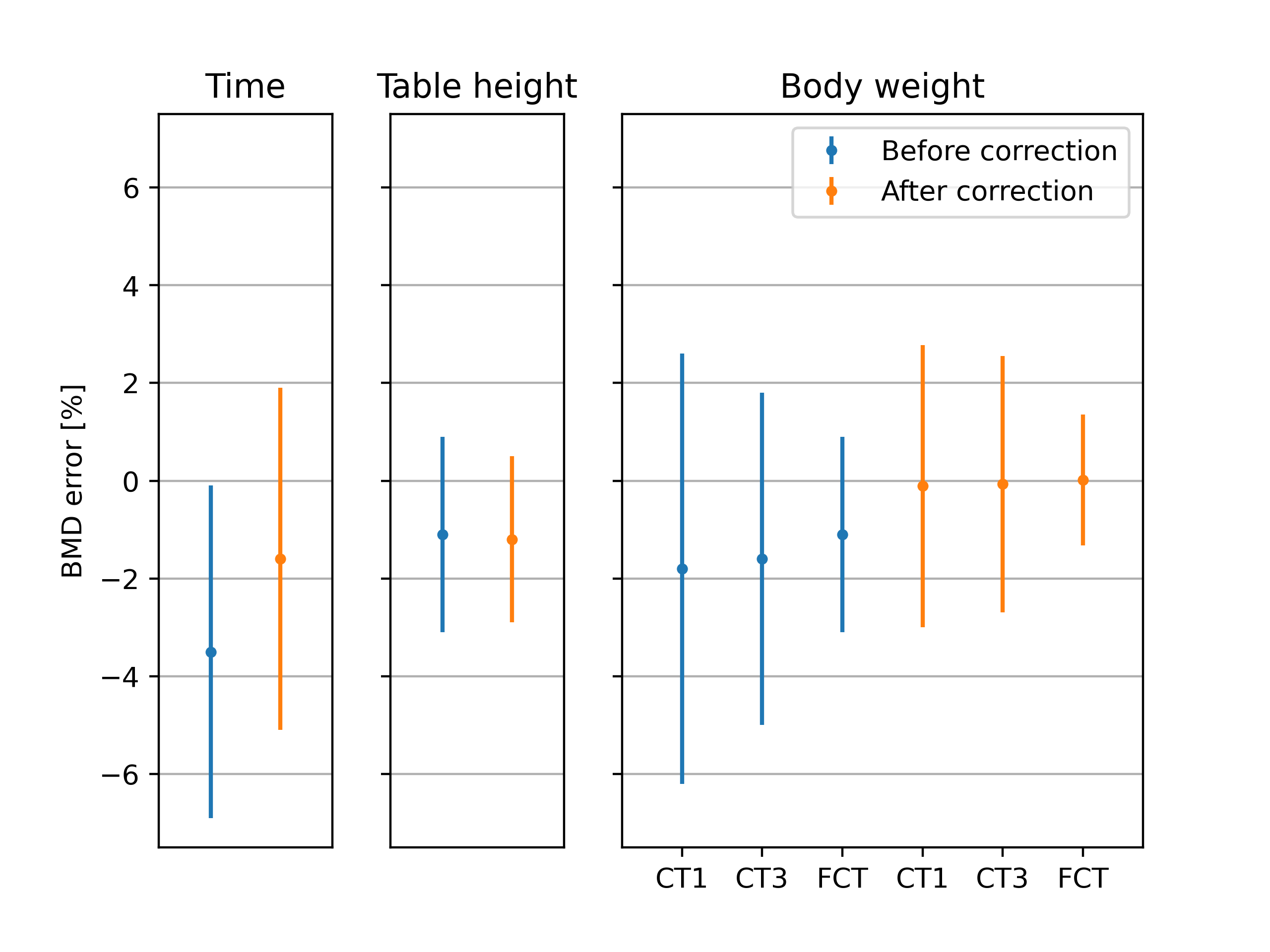


Supplementary Figure 4: Effect of the correction methods on the BMD error.
